# Development and Validation of a Multivariate Diagnostic Model for Major Depressive Disorder With Comorbid Insomnia Based on Lymphocyte Subsets and Resting-State Functional MRI

**DOI:** 10.1155/da/4530547

**Published:** 2025-11-30

**Authors:** Guangyuan Xia, Yongxue Hu, Jingyu Shi, Xue Shen, Peifan Li, Lei Zheng, Fangxian Chai, Yiming Wang, Xingde Liu

**Affiliations:** ^1^Department of Psychiatry, The Affiliated Hospital of Guizhou Medical University, Guiyang 550004, Guizhou province, China; ^2^Department of Neurology, The First Affiliated Hospital of Guizhou University of Traditional Chinese Medicine, Guiyang 550001, Guizhou province, China; ^3^Department of Clinical Laboratory, The Affiliated Hospital of Guizhou Medical University, Guiyang 550004, Guizhou province, China; ^4^Guizhou Medical University, Guiyang 550025, Guizhou province, China; ^5^Department of Cardiology, The Second Affiliated Hospital of Guizhou University of Traditional Chinese Medicine, Guiyang 550003, Guizhou province, China

**Keywords:** fusiform gyrus, insomnia disorder, lingual gyrus, lymphocyte subsets, major depressive disorder

## Abstract

**Objective:**

This study aimed to investigate the relationship between alterations in lymphocyte subsets and resting-state functional magnetic resonance imaging (rs-fMRI) patterns in patients with comorbid major depressive disorder (MDD) and insomnia disorder (ID).

**Methods:**

A total of 114 patients with MDD, 108 with ID, 126 with comorbid MDD and ID, and 168 healthy controls (HCs) were recruited, all experiencing their first episode. Emotional and sleep quality were assessed using the 17-item Hamilton Depression Rating Scale (HAMD-17), self-rating depression scale (SDS), Hamilton Anxiety Scale, self-rating anxiety scale (SAS), Pittsburgh Sleep Quality Index (PSQI), and Insomnia Severity Index (ISI). rs-fMRI data and lymphocyte subsets were analyzed. Multivariate prediction models were constructed using correlation analysis, least absolute shrinkage and selection operator (LASSO) regression with 10-fold cross-validation, and logistic regression. Model performance was evaluated with calibration curves and receiver operating characteristic (ROC) analysis.

**Results:**

No significant differences were observed in age (*p*=0.552), sex distribution (*p*=0.248), education level, or anxiety scores among the four groups, whereas depression and insomnia scores differed significantly (all *p*  < 0.0001). The MDD with comorbid insomnia (iMDD) group exhibited lower fractional amplitude of low-frequency fluctuations (fALFFs) in the right lingual gyrus and fusiform gyrus compared to the MDD, ID, and HC groups. Additionally, compared with HCs, CD3^+^ and CD4^+^ T cell percentages were elevated, while natural killer (NK) cell percentage was reduced, with the most pronounced alterations in the iMDD group. fALFF values were negatively correlated with CD3^+^ and CD4^+^ T cell percentages, but positively correlated with NK cell percentage. The fALFF in the right lingual gyrus, CD4^+^ T and NK cell percentage, SDS score, and ISI score were identified as key risk predictors. Multivariable prediction models for ID, MDD, and iMDD demonstrated robust calibration (e.g., calibration degree = 0.502), high discrimination (AUC for iMDD vs. HC = 0.991; MDD vs. ID = 0.821), and good clinical applicability.

**Conclusions:**

The identified risk predictors might facilitate individualized clinical decision-making for iMDD patients. While the multivariable prediction model demonstrated strong internal diagnostic accuracy, further external validation using independent cohorts is needed to confirm its generalizability.

## 1. Introduction

Mental health is increasingly recognized as a global concern, with major depressive disorder (MDD) and its comorbidity, Insomnia disorder (ID), posing significant public health challenges [1]. According to projections by the World Health Organization, MDD is expected to become the leading contributor to the global burden of disease by 2030 [2, 3]. Current techniques available for MDD diagnosis mostly rely on subjective reports of symptoms and clinical assessments, which have poor identification rates and uncertain treatment outcomes [4, 5]. A bidirectional relationship between MDD and ID has been well established, with evidence indicating that 50%–90% of patients experience MDD comorbid insomnia, often presenting as early-morning awakening [6]. Persistent ID occurring up to 12 months before the onset of MDD has been shown to significantly increase the risk of progression to MDD [7]. Conversely, individuals with insomnia face a significantly increased risk of developing subsequent depression. A meta-analysis has indicated that nondepressed individuals with insomnia are twice as likely to develop MDD compared to those without sleep difficulties [8]. Despite the critical role this comorbidity plays in treatment failures and poor prognosis, our understanding of MDD with comorbid insomnia (iMDD) remains limited.

The MDD and ID development is primarily attributed to the interaction between the immune system and the nervous system [9]. Changes in lymphocyte subpopulations suggest involvement of the immune system in the development of MDD and ID [10, 11]. Several studies have examined changes in lymphocyte subpopulations among patients with MDD; however, the reported findings remain inconsistent [12–14]. Other studies have demonstrated that sleep plays a crucial role in immune function, with sleep deprivation impairing the immune system's ability to defend against pathogens [15]. Dinges et al. [16] have highlighted the crucial role of natural killer (NK) cell function within lymphocyte subpopulations in mediating the immune response to sleep deprivation. Electroencephalographic (EEG) assessments comparing individuals with iMDD to healthy controls (HCs) demonstrated a positive correlation between sleep duration and NK cell function [17]. Moreover, sleep quality showed a positive correlation with CD4^+^ T cell levels within lymphocyte subpopulations, whereas chronic insomnia was associated with reduced CD4^+^ T cell levels [18]. Advances in understanding immune system functioning hold significant potential for improving the prevention and treatment of psychiatric disorders, including MDD and ID. However, a substantial gap remains in fully elucidating their manifestations and underlying mechanisms in iMDD patients.

MDD is characterized by diverse manifestations and complex pathogenic mechanisms, involving both brain and mind factors [19–21]. Univariate approaches are limited in their ability to identify biomarkers and elucidate the pathological mechanisms of iMDD, as they fail to adequately account for the multifactorial and complex nature of disease development. Developing predictive models that integrate multiple data types for iMDD diagnosis has become a key focus of current research. Recent studies have combined functional imaging markers with immune system function indicators to enhance diagnostic accuracy and mechanistic understanding. Resting-state functional magnetic resonance imaging (rs-fMRI) is a powerful tool for evaluating brain function in patients with psychiatric disorders such as MDD, providing valuable insights into early disease characteristics [22]. Changes in immune system function are reportedly associated with disruptions in neural activity in regions involved in emotion processing, regulation, and executive control [23, 24]. Meanwhile, monocytes aggregate toward the prefrontal regulatory system in the brain, potentially related to MDD and stress-related diseases [25]. However, the potential of combining functional imaging indicators with immune system function indicators has not been fully realized [26].

This study aimed to explore biomarkers suitable for early diagnosis of iMDD by combining advanced rs-fMRI technology and lymphocyte subpopulation analysis. Through fractional amplitude of low-frequency fluctuations (fALFFs) analyses, we investigated functional changes in various brain regions of iMDD patients, analyzed clinical symptoms associated with rs-fMRI abnormalities, and provided rs-fMRI indicators helpful for iMDD patients based on the symptoms. Further, flow cytometry was conducted to detect characteristic changes in lymphocyte subpopulations of iMDD patients, to elucidate the potential connections between lymphocyte subpopulations and iMDD. Finally, we established a multidimensional predictive model to explore objective indicators suitable for early diagnosis of iMDD patients, providing more accurate references for clinical diagnosis, treatment selection, and prognosis assessment.

Distinguishing iMDD from MDD alone is clinically critical as the comorbid condition is associated with greater severity of depression, poorer response to standard antidepressants, increased risk of suicide, and a more chronic course of illness, necessitating different treatment strategies that address both mood and sleep pathologies simultaneously.

A diagnostic model integrating objective biological parameters from rs-fMRI (reflecting central neural dysfunction) and lymphocyte subsets (reflecting peripheral immune dysregulation) has the potential to be more accurate because it captures the core pathophysiological pathways of iMDD, moving beyond reliance on subjective symptoms alone. This multimodal approach offers a more robust and biologically grounded basis for diagnosis.

## 2. Materials and Methods

### 2.1. Study Design and Participant Recruitment

This single-center diagnostic model development study recruited patients presenting with primary complaints of depression or insomnia at the psychiatric outpatient clinic of the Affiliated Hospital of Guizhou Medical University between July 2020 and October 2021. All participants were diagnosed with initial-onset depressive disorder or ID based on the Diagnostic and Statistical Manual of Mental Disorders, 5th edition (DSM-5), as confirmed by two experienced psychiatrists. A priori sample size estimation was conducted using G^*∗*^Power software. Assuming a medium effect size (*f* = 0.25), an alpha error of 0.05, and a desired power of 0.95 for one-way analysis of variance (ANOVA) across four groups, the required minimum sample size was calculated to be 356. The final enrolled sample consisted of 516 participants, exceeding the required threshold.

According to DSM-5 criteria, a total of 114 patients with MDD (51 males and 63 females), 108 patients with ID (51 males and 57 females), and 126 patients with iMDD (57 males and 69 females) were enrolled, all of whom had experienced their first episode. Additionally, 168 HCs (66 males and 102 females) were recruited from the physical examination center of the same hospital during the same period. The HC group was matched with the patient groups by sex, educational background, and age, ensuring no significant differences in these baseline demographic characteristics (Table 1).

### 2.2. Emotional State and Sleep Quality Assessments

Emotional state was evaluated using the Hamilton Depression Rating Scale-17 (HAMD-17), the self-rating depression scale (SDS), the Hamilton Anxiety Rating Scale (HAMA), and the self-rating anxiety scale (SAS). Sleep quality was assessed with the Pittsburgh Sleep Quality Index (PSQI) and the Insomnia Severity Index (ISI). All assessments were conducted and scored by qualified psychiatrists on the same day as the magnetic resonance imaging (MRI) scans.

### 2.3. Inclusion and Exclusion Criteria

Participants with MDD were included if they met *DSM-5* criteria confirmed by two attending psychiatrists. The inclusion criteria included HAMD-17 > 17, SDS > 62, HAMA < 14, SAS < 50, PSQI < 7, ISI < 8, no use of psychotropic or related drugs within the previous 6 months, no history of manic episodes, aged 18–55 years, right-handed, and possessing at least a junior high school education. ID participants were required to meet DSM-5 criteria for insomnia, with insomnia complaints, HAMD-17 < 7, SDS < 53, HAMA < 14, SAS < 50, PSQI ≥ 7, ISI ≥ 8, and the same general criteria as the MDD group. The iMDD group included patients with comorbid insomnia and MDD, who met DSM-5 criteria, HAMD-17 > 17, SDS > 62, HAMA < 14, SAS < 50, PSQI ≥ 7, ISI ≥ 8, and the same general requirements as the other groups. HCs were required to have good mood and sleep quality, with no psychiatric diagnosis, HAMD-17 < 7, SDS < 53, HAMA < 14, SAS < 50, PSQI < 7, ISI < 8, no personal or family psychiatric history, and to meet the same general criteria as the patient groups.

Exclusion criteria for all patient groups included organic brain disease, severe craniocerebral trauma, major physical illness, recent infection, intellectual disability, substance abuse, family psychiatric history, MRI contraindications, or pregnancy/lactation. HCs were additionally excluded for psychiatric disorders or hereditary mental illness.

### 2.4. rs-fMRI Data Acquisition

Diagnosis, clinical assessment, and rs-fMRI data were obtained on the same day. Participants refrained from using psychotropic, analgesic, or narcotic medications prior to data collection to avoid confounding effects. Before scanning, participants were informed of the procedures and safety precautions. During imaging, they were required to be in a supine position with the head comfortably stabilized using a sponge pad to minimize both voluntary and involuntary movements. Sponge earplugs were provided to reduce the impact of scanner noise. Participants were instructed to lie still, maintain normal breathing with eyes closed, remain conscious, and avoid engaging in specific thoughts. rs-fMRI data were acquired using an Achieva 3.0T MRI scanner (Philips, Netherlands) equipped with a SENSE-XL-Torso 8-channel cranial coil, operated by trained professional technicians [27, 28].

### 2.5. rs-fMRI Data Analysis

Prior to preprocessing, all images were reviewed by professional radiologists to confirm the absence of significant artifacts or structural deformations. Raw DICOM-formatted MRI data were converted to NIFTI format using dcm2niigui. Preprocessing of the neuroimaging data was conducted using SPM12 and Restplus software within MATLAB (R2013b) [29]. The fALFFs were calculated following the methodology described by Shu et al. [30].

### 2.6. Peripheral Blood Lymphocyte Subset Assessment

Between 7:00 and 9:00 a.m. on the day following enrollment, 3 mL of fasting venous blood was collected from each participant into EDTA-K2 anticoagulant tubes. Samples were gently mixed and processed for lymphocyte subset analysis using flow cytometry (BD FACSCanto, USA). Sample processing and the establishment of the gating strategy for lymphocyte subsets were performed according to the method described by Jha et al. [31]. Using the CD45/SSC gating strategy, CD45 was plotted on the *x*-axis and side scatter (SSC) on the *y*-axis, with CD45^+^ SSC^low^ defined as the lymphocyte population on the CD45/SSC dot plot. Within this lymphocyte gate, the proportion of CD3^+^ T cells among total lymphocytes was determined. The percentages of CD4^+^ and CD8^+^ T cells were calculated within the CD3^+^ T cell gate. The proportions of B cells and NK cells were assessed within the CD3^−^ lymphocyte population.

### 2.7. Multivariable Prediction Model Construction for Depressive Disorder and Comorbid Insomnia

Based on rs-fMRI results, lymphocyte subset proportions, and clinical assessment scores, correlations among variables were analyzed using the R corrplot package. Potential risk predictors were identified through least absolute shrinkage and selection operator (LASSO) regression with 10-fold cross-validation. Logistic regression models were then constructed using the selected predictors and expressed as regression equations. These models were visualized using nomogram plots, which were further used to calculate the predicted probabilities for MDD, ID, and iMDD diagnoses. Model performance was evaluated by assessing calibration curves for goodness-of-fit and receiver operating characteristic (ROC) curves to determine discriminatory ability.

### 2.8. Statistical Analysis

Data were analyzed using SPSS 23.0 and DecisionLinnc 1.0 [32]. Normality and homogeneity of variance were assessed for all variables. For results meeting the assumptions of normality and equal variance, one-way ANOVA was applied. Variables that did not meet these assumptions were analyzed using the Kruskal–Wallis test. Differences in sex distribution across the four groups were evaluated using the chi-square test, with statistical significance set at *p*  < 0.05.

## 3. Results

### 3.1. Comparison of Baseline Characteristics Among Study Groups

Statistical analyses revealed no significant differences in age (*p*=0.552), years of education (*p*=0.805), sex distribution (*p*=0.248), HAMA (*p*=0.162), or SAS scores (*p*=0.641) among the four groups. In contrast, significant differences were observed in Hamilton Depression Rating Scale-17 (HAMD-17), SDS, PSQI, and ISI scores (*p* < 0.0001). Specifically, the MDD and iMDD groups exhibited higher HAMD-17 and SDS scores compared with the HC and ID groups, while the ID and iMDD groups demonstrated elevated PSQI and ISI scores relative to the HC and MDD groups (*p* < 0.0001), as shown in Table 1. These findings suggest that depressive symptom severity predominantly distinguished the MDD and iMDD groups, whereas sleep disturbance was more characteristic of the ID and iMDD groups.

### 3.2. Comparison of fALFF in the Right Lingual and Fusiform Gyri Across HC, MDD, ID, and iMDD Groups

**Figure 1:**
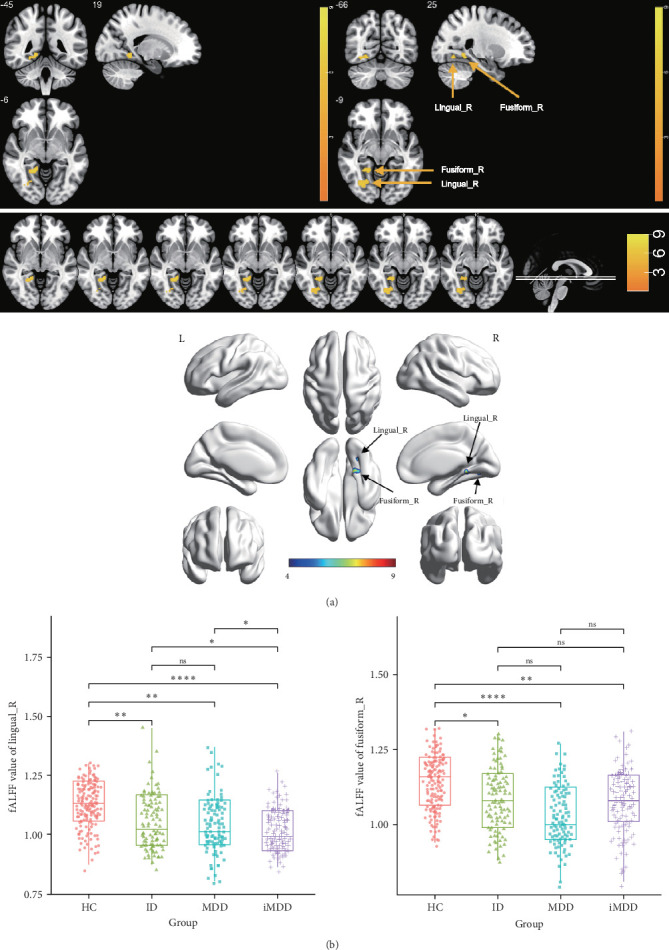
Comparison of fractional amplitude of low-frequency fluctuations (fALFFs) values in the right lingual and fusiform areas. (A) Comparison by resting-state functional magnetic resonance imaging (rs-fMRI). The yellow and blue represent the brain regions with different fALFF values. Lingual_R, the right lingual gyrus; Fusiform_R, the right fusiform gyrus. (B) Comparison by rank sum test. *⁣*^*∗*^*p*  < 0.05; *⁣*^*∗∗*^*p*  < 0.01; *⁣*^*∗∗∗∗*^*p*  < 0.0001; ns, nonsignificant.

Significant differences in fALFF values within the right lingual gyrus and fusiform gyrus were observed among the HC, MDD, ID, and iMDD groups (*p* < 0.01, FDRc = 29) (Table 2, Figure 1A). Compared with the HC group, fALFF values in the right lingual gyrus were significantly reduced in the MDD (*p* < 0.01), ID (*p* < 0.01), and iMDD (*p* < 0.0001) groups. Moreover, the iMDD group exhibited a more pronounced reduction compared with both the MDD and ID groups (*p* < 0.05), while no significant difference was detected between the MDD and ID groups (*p* > 0.05) (Figure 1B). In the right fusiform gyrus, fALFF values were also markedly reduced in the ID (*p* < 0.05), MDD (*p* < 0.0001), and iMDD (*p* < 0.01) groups compared with HC. However, no significant differences were observed among the MDD, ID, and iMDD groups (*p* > 0.05).

### 3.3. Comparative Analysis of CD3^+^, CD4^+^, CD8^+^ T Cells, B Cells, and NK Cells Across HC, MDD, ID, and iMDD Groups

**Figure 2:**
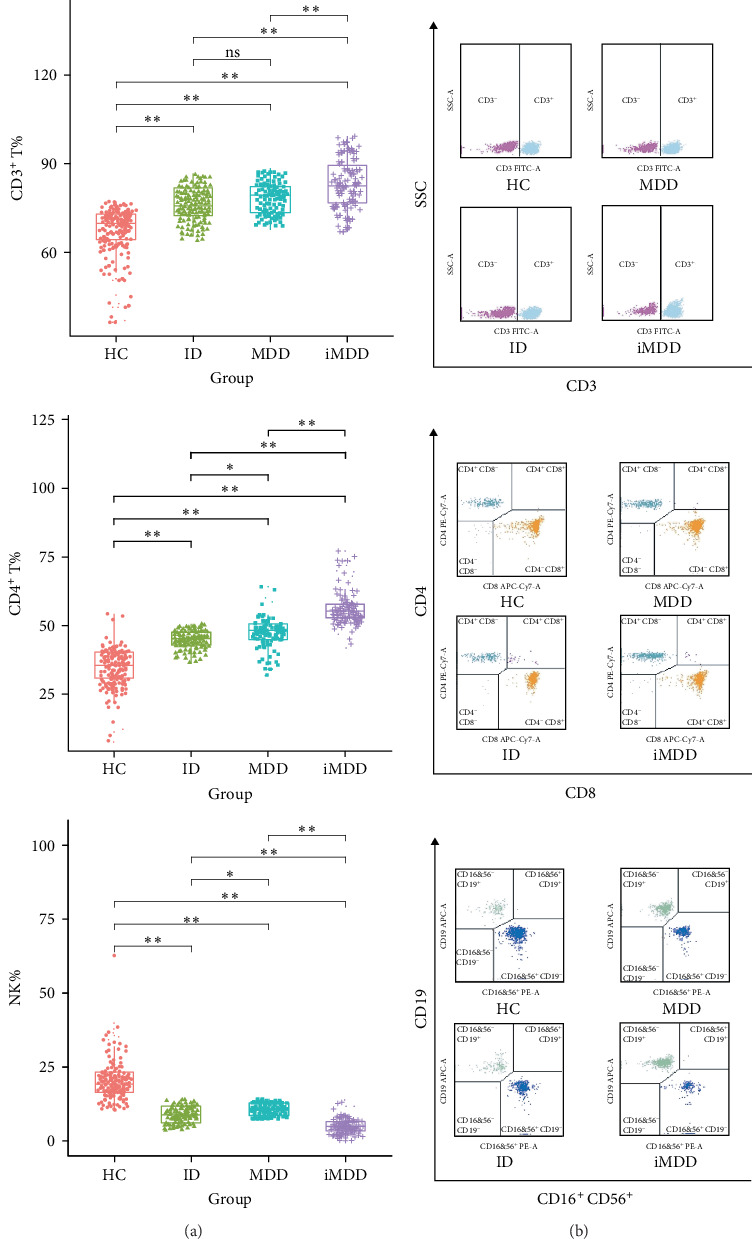
Distribution and group differences of peripheral blood lymphocyte subsets. (A) Comparison of CD3^+^ T cell, CD4^+^ T cell, and natural killer (NK) cell percentage by one-way analysis of variance (ANOVA). (B) Flow cytometry scatter plot. The scatter plot illustrates the molecular complexity of particles inside cells, dividing lymphocytes into CD3^+^ and CD3^−^ groups based on CD3 antigen molecule intensity, and identifies CD19 as the marker for B cells and CD16^+^ CD56^+^ as the phenotype of NK cells. *⁣*^*∗*^*p* < 0.05; *⁣*^*∗∗*^*p* < 0.01; ns, non-significant.

One-way ANOVA revealed significant differences in the percentages of CD3^+^ T cells, CD4^+^ T cells, and NK cells among the HC, MDD, ID, and iMDD groups (*p* < 0.0001), whereas no significant differences were observed for CD8^+^ T cells and B cells (*p* > 0.05) (Table 3). Compared with the HC group, the MDD, ID, and iMDD groups exhibited significant increases in CD3^+^ and CD4^+^ T cell percentages, accompanied by a marked reduction in NK cells (*p* < 0.01). Within the iMDD group, CD3^+^ and CD4^+^ T cell percentages were further elevated, while NK cell percentages were significantly decreased relative to both the MDD and ID groups (*p* < 0.01) (Figure 2A,B). Additionally, the MDD group showed a significant increase in CD4^+^ T cells compared with the ID group (*p* < 0.05), whereas the ID group demonstrated a greater reduction in NK cells relative to the MDD group (*p* < 0.05).

### 3.4. Correlation Analysis Between fALFF Measures and Lymphocyte Subsets

**Figure 3:**
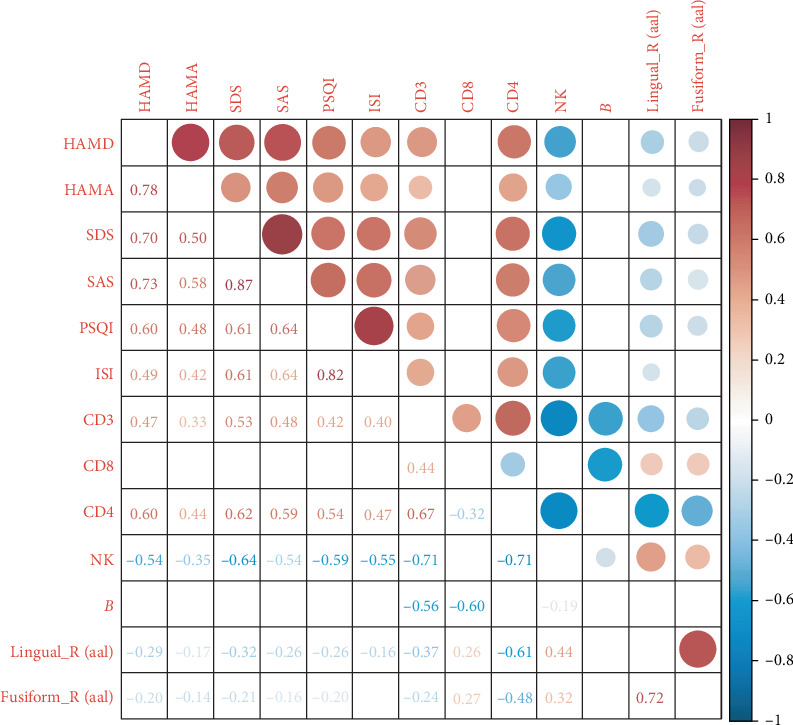
Correlations among rs-fMRI indices, lymphocyte subsets, and clinical scores. Red circle and numbers represent positive correlation; blue circle and numbers represent negative correlation. The depth of color reflects the significance of the correlation, with darker shades indicating stronger relationships. Circle size corresponds to the magnitude of the correlation.

Correlation analysis of the MDD, ID, and iMDD groups across 13 variables revealed significant associations between fALFF values and lymphocyte subsets. Specifically, fALFF in the right lingual gyrus was negatively correlated with the percentages of CD3^+^ T cells (*r* = –0.37) and CD4^+^ T cells (*r* = –0.61), while showing a positive correlation with NK cells (*r* = 0.44) (*p* < 0.05). Similarly, fALFF in the right fusiform gyrus demonstrated negative correlations with CD3^+^ T cells (*r* = –0.24) and CD4^+^ T cells (*r* = –0.48), and a positive correlation with NK cells (*r* = 0.32) (*p* < 0.05). Among the characteristic indicators, the fALFF of the right lingual gyrus exhibited particularly strong associations with CD4^+^ T cells and NK cells (Figure 3).

### 3.5. Screening and Selection of Risk Predictors for Multivariate Prediction Models

At λ.1se = 0.01650165, 10 candidate risk predictors were identified for constructing multivariate prediction models for MDD, ID, and iMDD. These included right lingual gyrus fALFF, right fusiform gyrus fALFF, CD3^+^ T cells, CD4^+^ T cells, NK cells, B cells, HAMD-17 score, SDS score, PSQI score, and ISI score. The significance of these predictors was further evaluated using the likelihood ratio test, with the Akaike information criterion (AIC) applied to assess model fit, where higher AIC values indicate stronger correlation with the model. Factors with high AIC values (*p* < 0.01) were selected as optimal predictors, resulting in five final variables: right lingual gyrus fALFF, CD4^+^ T cells, NK cells, SDS score, and ISI score (Table 4).

### 3.6. Construction of Multivariable Prediction Models and Diagnostic Nomograms

**Figure 4:**
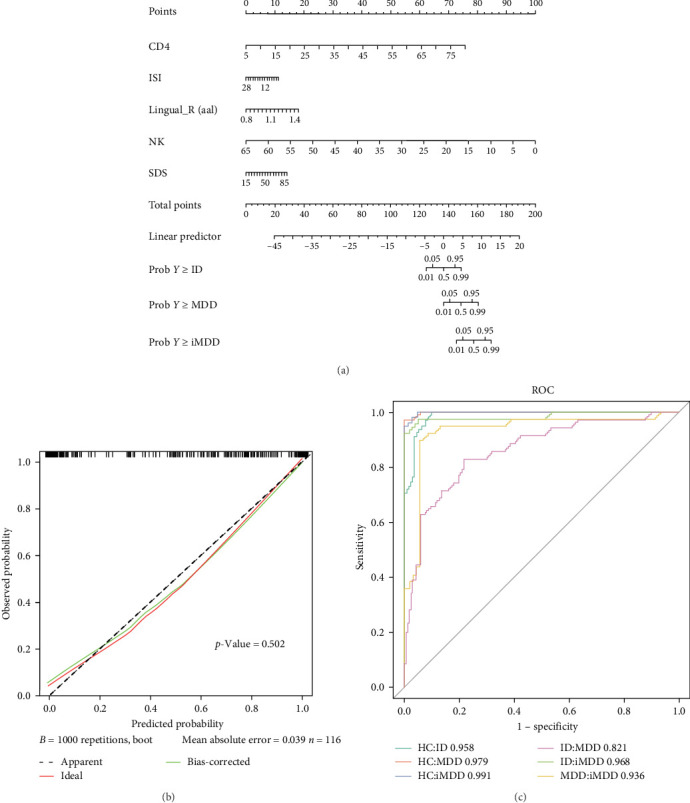
Construction and validation of the multivariate predictive model for MDD, ID, and iMDD. (A) Nomogram plot of multivariable prediction models. Points: the values corresponding to the risk predictors, CD4: CD4^+^ T cell percentage, ISI: Insomnia Severity Index score, Lingual_R (aal): right lingual area fALFF value, NK: NK cell percentage, SDS: self-rating depression scale score, total points: the total sum of points scores for the five risk predictors, linear predictor: the linear prediction value. Prob *Y* > = ID, probability interval of diagnosis of ID; prob *Y* > = MDD, probability interval of diagnosis of MDD; and prob *Y* > = iMDD, probability interval for diagnosis of iMDD. (B) Calibration curves of multivariable prediction models. (C) Receiver operating characteristic (ROC) curves of multivariable prediction models. The area under the curve (AUC) of ROC varies between 0.5 and 1.0, with values greater than 0.75 indicating good discrimination, greater than 0.9 signifying very good discrimination, and equal to 1.0 demonstrating perfect discrimination. *⁣*^*∗*^*p*  < 0.05; *⁣*^*∗∗*^*p*  < 0.01; ns, nonsignificant.

The logistic regression analysis (LRA) results of the multivariable prediction models based on selected risk predictors are presented in Table 5. Using LRA, a nomogram was constructed to facilitate the diagnosis of MDD, ID, and iMDD. In the nomogram (Figure 4A), each risk predictor was assigned a corresponding point value, and the total score was calculated as the sum of these points. The diagnostic probabilities for ID, MDD, and iMDD were then obtained by projecting a vertical line from the total score to the probability scale.

For example, a patient with a CD4^+^ T cell percentage of 45, ISI score of 12, right fALFF value of 1.4, NK cell percentage of 15, and SDS score of 50 received point allocations of 40, 10, 20, 76, and 10, respectively, yielding a total score of 156. Referring to the Total Points scale, this score corresponded to an estimated probability of ~95% for MDD and 50% for iMDD, while no probability interval was reached for ID, indicating a diagnosis of MDD. Similarly, another patient with a total score of 168 was assigned an estimated probability of 99% for iMDD, with no diagnostic probability for ID or MDD. In cases where the total score did not intersect with any probability interval, the model did not provide a diagnostic prediction.

### 3.7. Validation of Multivariate Prediction Models

Calibration curves demonstrated a calibration degree of 0.502, indicating high predictive accuracy and substantial overlap among MDD, ID, and iMDD models (Figure 4B). ROC analysis confirmed model performance, with AUCs of 0.991 for HC vs. iMDD, 0.821 for ID vs. MDD, and >0.9 for all other comparisons, reflecting excellent discriminative ability across cohorts (Figure 4C, Table 6).

## 4. Discussion

Lingual gyrus, situated between the fissura calcarina and collateral sulcus, plays a crucial role in visual information processing and facial emotion recognition [33, 34]. Dysfunction in the lingual gyrus can heighten the visual area's response to sad faces, potentially impacting cognitive processing [35]. Additionally, the volume of the lingual gyrus is associated with antidepressant reactivity, and its size may serve as a predictor of treatment response in individuals with MDD [36]. A study has demonstrated a reduction in the amplitude of low-frequency fluctuations (ALFFs) within the right lingual gyrus among MDD individuals, indicating that abnormal lingual gyrus function might contribute to the cognitive impairments observed in these patients [37]. Adjacent to the lingual gyrus, the fusiform gyrus is part of the same visual–cognitive network. Reduced fALFF values in the right lingual and fusiform areas have been observed in MDD individuals, suggesting that these regions might be potential targets for therapeutic intervention [9]. Furthermore, fALFF values in the fusiform gyrus have been reduced, and functional connectivity (FC) has been weakened in patients with ID. Notably, enhanced FC of the fusiform gyrus has been observed following cognitive behavioral therapy, underscoring its key role in the neuropathology of insomnia [38, 39].

In this study, fALFF values in the lingual and fusiform gyri were significantly reduced in the MDD, ID, and iMDD groups compared to HC, indicating impaired intrinsic neural activity in these regions, consistent with previous findings [9, 38, 40, 41]. An apparent reduction was observed in the iMDD group, suggesting a superimposed impact on the functions of the right lingual gyrus and fusiform gyri in these patients. However, the underlying mechanism of this superimposition remains unclear and warrants further investigation. No significant differences were observed in fALFF measurements of the fusiform gyrus across the ID, MDD, and iMDD cohorts, indicating that MDD and ID might exert distinct, nonoverlapping effects on right fusiform gyrus function. Further studies are warranted to investigate cognitive performance in iMDD patients and to elucidate the relationship between rs-fMRI alterations and cognitive function.

The intricate connection between psychiatric disorders and dysregulation of lymphocyte subsets serves as a pivotal biological element in the development of both MDD and ID [42]. The percentages of CD3^+^ and CD4^+^ T cells were significantly elevated, whereas NK cell levels were markedly reduced in the MDD, ID, and iMDD cohorts compared to HCs, consistent with previous reports [43, 44]. T cells, particularly CD3^+^ T cells, played a critical role in mediating the link between immune alterations and MDD. Reduced T-cell reactivity contributed to immune dysregulation in affected patients [45]. Tryptophan and 5-hydroxytryptamine (5-HT) were also involved in modulating cell-mediated immune responses in MDD. Effector T-cell proliferation depended on tryptophan, which, in the context of MDD, was depleted by proinflammatory cytokines or stressful conditions, ultimately contributing to T-cell dysfunction [46]. 5-HT influenced the activity of macrophages, NK cells, and T cells, while T-cell function, in turn, modulated the release of 5-HT [47]. Sleep exerts a critical influence on immune system function, and insufficient sleep disrupts both the number and activity of immune cells, particularly T lymphocytes [43]. In addition, adolescents who slept less than 8 h exhibited elevated counts of monocytes, white blood cells, neutrophils, and T-lymphocyte subsets [48]. CD4^+^ T cells secreted a range of cytokines and were elevated in individuals with MDD, suggesting their potential utility as a biomarker for the disorder [49, 50]. However, the percentage of CD4^+^ T cells in patients with MDD was not considered fixed. Christoffersson et al. [51] reported that CD4^+^ T cell counts in the spleen decreased following sleep deprivation. These inconsistencies may be explained by variations in MDD severity and duration, depressive subtypes, and patients' pharmacological treatment histories. Moreover, NK cell activity has been linked to antidepressant response, and its reduction further supports an association between MDD and increased susceptibility to infectious diseases [52, 53]. Circulating NK cells may serve as clinically relevant predictors of antidepressant treatment response, and their decreased activity suggests a potential link between MDD and increased vulnerability to infectious diseases [45]. Furthermore, NK cell activity plays a pivotal role in the immune response to sleep deprivation, increasing with sufficient sleep and decreasing under conditions of insufficient sleep [54, 55]. The reduction in NK cell activity was associated with the severity of initial insomnia in patients with MDD [56], and sleep parameters were positively associated with NK cell activity [17].

Elucidating the interaction between immune function and brain activity may contribute to more personalized diagnosis and treatment of patients with MDD, and enhance the efficacy of antidepressant therapies through the development of immunomodulation-based strategies [57]. In our study, we identified significant alterations in the percentages of CD3^+^ and CD4^+^ T cells, along with a reduction in NK cell percentage in the iMDD group compared to the MDD and ID groups. These findings suggested a potential superimposed effect of iMDD on CD3^+^ and CD4^+^ T cells. Further cohort studies and interventional trials are needed to clarify this relationship. Moreover, incorporating immunoregulatory therapy alongside antidepressant treatment may represent a promising avenue to improve outcomes in iMDD patients exhibiting immune alterations. Correlation analyses between rs-fMRI and lymphocyte subsets in patients with MDD, ID, and iMDD demonstrated negative associations between fALFF values in the right lingual and fusiform gyri with CD3^+^ and CD4^+^ T cell percentages, while positive correlations were observed with NK cells. These findings suggested a potential link between neural activity patterns and immune alterations. Notably, brain regions such as the anterior cingulate cortex (ACC) and medial prefrontal cortex (mPFC), which are central to psychological and behavioral regulation, may influence immune function through sympathetic and neuroendocrine pathways [58]. These pathways regulate the expression of inflammatory genes and coordinate immune responses that are closely integrated with physiological and psychological processes essential for survival [59]. Theoretically, the functions of the ACC and mPFC may be altered during the regulation of central inflammation by immune cells, potentially contributing to the pathological mechanisms underlying the observed association between rs-fMRI findings and lymphocyte subset alterations in iMDD patients [60]. However, causal relationships between rs-fMRI alterations and iMDD have not yet been established, and longitudinal studies are required to further elucidate these characteristic changes. Such investigations could provide critical insights into disease mechanisms and facilitate the development of personalized diagnostic and therapeutic strategies. Multivariate prediction models developed using LASSO regression have demonstrated usefulness in identifying high-risk groups, informing preventive strategies, and improving clinical outcomes in patients with MDD and ID [61]. LASSO regression analysis identified the fALFF value of the right lingual gyrus, CD4^+^ T cell percentage, NK cell percentage, SDS score, and ISI score as optimal risk predictors for MDD, ID, and iMDD. These predictors were incorporated into a multivariate prediction model, which demonstrated good calibration and high diagnostic accuracy (calibration degree = 0.502, *p*  > 0.05). The integration of multiple biological parameters from distinct physiological systems, including neuroimaging markers reflecting central neural dysfunction and peripheral immune indicators, offers a more robust approach to precision diagnosis, moving beyond univariate biomarkers alone [62]. Analysis of MDD-related risk predictors enhanced understanding of the disorder and contributed to the development of targeted prevention strategies. The influence of one or more risk predictors under specific environmental conditions could precipitate depressive episodes, whereas timely intervention might help prevent the worsening of MDD symptoms [63]. Incorporating rs-fMRI as a risk predictor might enhance diagnostic accuracy and improve treatment outcomes in MDD. Furthermore, immunological predictors should be integrated into personalized diagnostic and therapeutic strategies, which may contribute to optimizing treatment efficacy [64]. We also developed nomogram plots, which allowed estimation of diagnostic prediction probabilities for MDD, ID, and iMDD by calculating scores corresponding to the identified risk predictors. The multivariate prediction models for MDD, ID, and iMDD developed in this study incorporated a comprehensive set of risk predictors, offering clinical utility and enhancing diagnostic accuracy. However, the models were limited to internal validation; future studies incorporating external validation with data from multiple medical institutions are necessary to further evaluate their generalizability.

This study has several limitations. First, the participants were recruited from a single medical center, which might limit the generalizability of our findings to broader populations. Second, the cross-sectional nature of the data precludes causal inferences. Future multicenter longitudinal studies are warranted to validate the predictive model externally and explore temporal changes in biomarkers.

## 5. Conclusion

Patients with MDD, ID, and insomnia comorbid with MDD (iMDD) exhibited functional impairments in the right lingual gyrus, accompanied by distinct alterations in peripheral blood lymphocyte subsets. iMDD might exert a cumulative effect on both right lingual gyrus function and lymphocyte subset profiles. Furthermore, rs-fMRI alterations in these patient groups were closely associated with changes in lymphocyte subsets. Notably, the fALFF value of the right lingual gyrus, along with CD4^+^ T cell and NK cell levels, might serve as potential biomarkers for identifying individuals at risk for MDD, ID, and iMDD.

## Figures and Tables

**Table 1:** Comparison of subject information.

Items	MDD (*n* = 114)	ID (*n* = 108)	iMDD (*n* = 126)	HC (*n* = 168)	*p*-Value
Age	30.55 ± 7.80	34.65 ± 11.65	30.25 ± 7.55	29.45 ± 11.10	0.552
Gender (male/female)	51/63	51/57	57/69	66/102	0.248
Education years	13.10 ± 2.20	14.15 ± 3.42	13.20 ± 2.00	16.58 ± 2.70	0.805
HAMD-17	31.40 ± 11.38	21.25 ± 10.20	35.22 ± 7.30	7.85 ± 4.50	<0.0001
HAMA	9.15 ± 7.10	8.80 ± 6.40	9.75 ± 5.50	7.70 ± 4.20	0.162
SDS	40.90 ± 10.30	23.05 ± 8.95	39.80 ± 9.35	11.20 ± 3.22	<0.0001
SAS	34.55 ± 8.50	33.80 ± 11.10	35.10 ± 8.90	33.35 ± 9.80	0.641
PSQI	8.00 ± 2.90	15.70 ± 2.80	15.90 ± 2.65	5.85 ± 1.42	<0.0001
ISI	5.90 ± 1.70	19.00 ± 4.30	18.90 ± 3.70	3.70 ± 2.20	<0.0001

Abbreviations: HAMA, Hamilton Anxiety Scale; HAMD-17, Hamilton Depression Scale-17; HC, healthy control; ID, insomnia disorder; iMDD, comorbid major depressive disorder and insomnia disorder; ISI, Insomnia Severity Index; MDD, major depressive disorder; PSQI, Pittsburgh Sleep Quality Index; SAS, self-rating anxiety scale; SDS, self-rating depression scale.

**Table 2:** Results of the fractional amplitude of low-frequency fluctuations (fALFFs) values in the right lingual gyrus and fusiform gyrus.

Brain region (AAL)	Brodmann area	MNI peak coordinate (mm)			*T* value	Cluster size
		*X*	*Y*	*Z*		
Right lingual gyrus	19	18	−45	−6	8.5177	29
Right fusiform gyrus	18	24	−66	−9	7.4005	20

Abbreviations: AAL, anatomical automatic labeling; MNI, Montreal Neurological Institute.

**Table 3:** Comparison of the lymphocyte subsets characteristics.

Lymphocyte subsets	MDD (*n* = 114)	iMDD (*n* = 126)	ID (*n* = 108)	HC (*n* = 168)	*F* value	*p*-Value
CD3^+^ T cell percentage	77.70 ± 5.25	83.35 ± 8.65	75.75 ± 6.45	67.00 ± 8.20	39.85	<0.0001
CD4^+^ T cell percentage	47.45 ± 5.90	56.60 ± 6.15	45.10 ± 3.50	35.40 ± 6.95	95.20	<0.0001
CD8^+^ T cell percentage	27.30 ± 8.75	24.90 ± 8.30	27.60 ± 7.50	27.75 ± 5.05	1.25	0.290
B cell percentage	11.50 ± 5.05	11.20 ± 8.00	14.90 ± 5.40	11.95 ± 7.50	2.25	0.085
Natural killer (NK) cell percentage	10.45 ± 2.20	5.00 ± 4.10	8.90 ± 3.15	21.05 ± 8.25	81.50	<0.0001

**Table 4:** Maximum likelihood ratio test (LRT) for risk predictors.

Item	Df	AIC	LRT	Pr (>chi)
CD3^+^ T cell percentage	1	133.1	4.05	0.044
CD4^+^ T cell percentage	1	215.00	85.50	<0.0001
Right fusiform gyrus fALFF value	1	133.50	4.05	0.044
B cell percentage	1	135.00	5.80	0.12
HAMD	1	132.00	2.60	0.017
ISI	1	138.00	7.80	0.0052
Right lingual gyrus fALFF value	1	165.00	35.00	<0.0001
NK cell percentage	1	198.00	68.50	<0.0001
PSQI	1	132.00	2.30	0.015
SDS	1	140.00	10.50	0.0012

*Note:* Pr (>chi), *p*-value.

Abbreviations: AIC, Akaike information criterion; Df, degrees of freedom.

**Table 5:** Logistic analysis of risk predictors.

Items	Coef	S.E.	Pr (>|*Z*|)	Pr	OR
*y* > = ID	−21.3415	2.6900	−7.9350	<0.0001	5.3887E-10
*y* > = MDD	−25.8654	2.9200	−8.8566	<0.0001	5.8454E-12
*y* > = iMDD	−29.3237	3.0800	−9.5200	<0.0001	1.8403E-13
CD4	0.3950	0.0390	10.1282	<0.0001	1.4850
ISI	−0.1570	0.0270	−5.8148	<0.0001	0.8550
Right lingual gyrus fALFF value	10.6882	1.4400	7.4222	<0.0001	44100.0000
NK cell percentage	−0.6020	0.0620	−9.7097	<0.0001	0.5480
SDS	0.0730	0.0130	5.6154	<0.0001	1.0760

*Note:* Coef, regression coefficient; Pr, *p*-value; *Z*, *Z*-value.

Abbreviations: OR: odds ratio; S.E., standard error.

**Table 6:** Results of specificity, sensitivity, and area under the curve (AUC) of the multivariate predictive models.

Items	Specificity	Sensitivity	AUC
HC:ID	0.935454545	0.960588235	0.958
HC:MDD	0.990000000	0.961428571	0.979
HC:iMDD	0.995000000	0.995000000	0.991
ID:MDD	0.780117647	0.810571429	0.821
ID:iMDD	0.960588235	0.964358974	0.968
MDD:iMDD	0.965428571	0.890435897	0.936

## Data Availability

All data generated or analyzed during this study are included in this article.

## Nomenclature

AAL: Anatomical automatic labeling
AIC: Akaike information criterion
ANOVA: Analysis of variance
Coef: Regression coefficient
Df: Degrees of freedom
fALFFs: Fractional amplitude of low-frequency fluctuations
HAMA: Hamilton Anxiety Rating Scale
HAMD-17: Hamilton Depression Scale-17
HCs: Healthy controls
ID: Insomnia disorder
iMDD: Comorbid major depressive disorder and insomnia disorder
ISI: Insomnia Severity Index
LRA: Logistic regression analysis
MDD: Major depressive disorder
MNI: Montreal Neurological Institute
mPFC: Medial prefrontal cortex
NK cell: Natural killer cell
OR: Odds ratio
Pr (>chi): *p*-Value
PSQI: Pittsburgh Sleep Quality Index
ROC: Receiver operating characteristic
rs-fMRI: Resting-state functional magnetic resonance imaging
SAS: Self-rating anxiety scale
SDS: Self-rating depression scale
S.E.: Standard error
Z: *Z* value
5-HT: 5-hydroxytryptamine.

## References

[1] Marti C. N., Kunik M. E., Choi N. G. (2021). The Reciprocal Relationship Between Depression and Disability in Low-Income Homebound Older Adults Following Tele-Depression Treatment. *International Journal of Geriatric Psychiatry*.

[2] Lépine J. P., Briley M. (2011). The Increasing Burden of Depression. *Neuropsychiatric Disease and Treatment*.

[3] Zhong B.-L., Ruan Y.-F., Xu Y.-M., Chen W.-C., Liu L.-F. (2020). Prevalence and Recognition of Depressive Disorders Among Chinese Older Adults Receiving Primary Care: A Multi-Center Cross-Sectional Study. *Journal of Affective Disorders*.

[4] Locher C., Koechlin H., Zion S. R. (2017). Efficacy and Safety of Selective Serotonin Reuptake Inhibitors, Serotonin-Norepinephrine Reuptake Inhibitors, and Placebo for Common Psychiatric Disorders Among Children and Adolescents: A Systematic Review and Meta-Analysis. *JAMA Psychiatry*.

[5] Ding Z., Chen J., Zhong B.-L., Liu C.-L., Liu Z.-T. (2025). Emotional Stimulated Speech-Based Assisted Early Diagnosis of Depressive Disorders Using Personality-Enhanced Deep Learning. *Journal of Affective Disorders*.

[6] Tsuno N., Besset A., Ritchie K. (2005). Sleep and Depression. *Journal of Clinical Psychiatry*.

[7] Betti L., Palego L., Giannaccini G. (2018). Depression, Insomnia and Atypical Antidepressants. *Frontiers in Clinical Drug Research - CNS and Neurological Disorders*.

[8] Baglioni C., Battagliese G., Feige B. (2011). Insomnia as a Predictor of Depression: A Meta-Analytic Evaluation of Longitudinal Epidemiological Studies. *Journal of Affective Disorders*.

[9] Yang C., Zhang A., Jia A. (2018). Identify Abnormalities in Resting-State Brain Function Between First-Episode, Drug-Naive Major Depressive Disorder and Remitted Individuals: A 3-Year Retrospective Study. *NeuroReport*.

[10] Beumer W., Gibney S. M., Drexhage R. C. (2012). The Immune Theory of Psychiatric Diseases: A Key Role for Activated Microglia and Circulating Monocytes. *Journal of Leukocyte Biology*.

[11] Toben C., Baune B. T. (2015). An Act of Balance Between Adaptive and Maladaptive Immunity in Depression: A Role for T Lymphocytes. *Journal of Neuroimmune Pharmacology*.

[12] Rahimi S., Peeri M., Azarbayjani M. A. (2020). Long-Term Exercise From Adolescence to Adulthood Reduces Anxiety- and Depression-Like Behaviors Following Maternal Immune Activation in Offspring. *Physiology & Behavior*.

[13] Maes M., Nani J. V., Noto C., Rizzo L., Hayashi M. A. F., Brietzke E. (2021). Impairments in Peripheral Blood T Effector and T Regulatory Lymphocytes in Bipolar Disorder are Associated With Staging of Illness and Anti-Cytomegalovirus IgG Levels. *Molecular Neurobiology*.

[14] Arreola R., Becerril-Villanueva E., Cruz-Fuentes C. (2015). Immunomodulatory Effects Mediated by Serotonin. *Journal of Immunology Research*.

[15] Akerstedt T., Ghilotti F., Schwarz J., Theorell-Haglöw J., Lindberg E. (2020). 0460 Insomnia In 400 Women: Polysomnography, Immune Parameters, Depression and Anxiety. *Sleep*.

[16] Dinges D. F., Douglas S. D., Zaugg L. (1994). Leukocytosis and Natural Killer Cell Function Parallel Neurobehavioral Fatigue Induced by 64 hours of Sleep Deprivation. *Journal of Clinical Investigation*.

[17] Irwin M., Smith T. L., Gillin J. C. (1992). Electroencephalographic Sleep and Natural Killer Activity in Depressed Patients and Control Subjects. *Psychosomatic Medicine*.

[18] El-Kader S. M. Abd, Al-Jiffri O. H. (2020). Aerobic Exercise Affects Sleep, Psychological Wellbeing and Immune System Parameters Among Subjects With Chronic Primary Insomnia. *African Health Sciences*.

[19] Germain A., Kupfer D. J. (2008). Circadian Rhythm Disturbances in Depression. *Human Psychopharmacology: Clinical and Experimental*.

[20] Abad V., Guilleminault C. (2009). Sleep and Psychiatry. *Zhurnal nevrologii i psikhiatrii imeni S.S. Korsakova*.

[21] Jialin A., Zhang H.-G., Wang X.-H. (2025). Cortical Activation Patterns in Generalized Anxiety and Major Depressive Disorders Measured by Multi-Channel Near-Infrared Spectroscopy. *Journal of Affective Disorders*.

[22] Gong Q., He Y. (2015). Depression, Neuroimaging and Connectomics: A Selective Overview. *Biological Psychiatry*.

[23] Tawakol A., Ishai A., Takx R. A. (2017). Relation Between Resting Amygdalar Activity and Cardiovascular Events: A Longitudinal and Cohort Study. *The Lancet*.

[24] Lasselin J., Treadway M. T., Lacourt T. E. (2017). Lipopolysaccharide Alters Motivated Behavior in a Monetary Reward Task: A Randomized Trial. *Neuropsychopharmacology*.

[25] Nusslock R., Brody G. H., Armstrong C. C. (2019). Higher Peripheral Inflammatory Signaling Associated With Lower Resting-State Functional Brain Connectivity in Emotion Regulation and Central Executive Networks. *Biological Psychiatry*.

[26] Lichtner G., Zacharias N., Spies C. D. (2021). Resting State Brain Network Functional Connectivity is Not Associated With Inflammatory Markers and Blood Cell Counts in Older Adults. *Clinical Neurophysiology*.

[27] Cho H. J., Jeong H., Park C. A., Son D. W., Shim S. Y. (2022). Altered Functional Connectivity in Children Born Very Preterm at School Age. *Scientific Reports*.

[28] Cui W., Zhang J., Xu F. (2021). MRI Evaluation of the Relationship Between Abnormalities in Vision-Related Brain Networks and Quality of Life in Patients With Migraine Without Aura. *Neuropsychiatric Disease and Treatment*.

[29] Yan C. G., Wang X. D., Zuo X. N., Zang Y. F. (2016). DPABI: Data Processing & Analysis for (Resting-State) Brain Imaging. *Neuroinformatics*.

[30] Shu Y., Kuang L., Huang Q., He L. (2020). Fractional Amplitude of Low-Frequency Fluctuation (fALFF) Alterations in Young Depressed Patients With Suicide Attempts After Cognitive Behavioral Therapy and Antidepressant Medication Cotherapy: A Resting-State fMRI Study. *Journal of Affective Disorders*.

[31] Jha M. K., Minhajuddin A., Gadad B. S., Trivedi M. H. (2017). Platelet-Derived Growth Factor as an Antidepressant Treatment Selection Biomarker: Higher Levels Selectively Predict Better Outcomes With Bupropion-SSRI Combination. *International Journal of Neuropsychopharmacology*.

[32] DecisionLinnc Core Team (2023). DecisionLinnc is a Platform That Integrates Multiple Programming Language Environments and Enables Data Processing, Data Analysis, and Machine Learning Through a Visual Interface. https://www.statsape.com/.

[33] Wang Y., Zhong S., Jia Y. (2015). Interhemispheric Resting State Functional Connectivity Abnormalities in Unipolar Depression and Bipolar Depression. *Bipolar Disorders*.

[34] Wu L., Wang C., Liu J. (2021). Voxel-Mirrored Homotopic Connectivity Associated With Change of Cognitive Function in Chronic Pontine Stroke. *Frontiers in Aging Neuroscience*.

[35] Keedwell P., Drapier D., Surguladze S., Giampietro V., Brammer M., Phillips M. (2009). Neural Markers of Symptomatic Improvement During Antidepressant Therapy in Severe Depression: Subgenual Cingulate and Visual Cortical Responses to Sad, but Not Happy, Facial Stimuli Are Correlated With Changes in Symptom Score. *Journal of Psychopharmacology*.

[36] Jung J., Kang J., Won E. (2014). Impact of Lingual Gyrus Volume on Antidepressant Response and Neurocognitive Functions in Major Depressive Disorder: A Voxel-Based Morphometry Study. *Journal of Affective Disorders*.

[37] Drevets W. C. (2007). Orbitofrontal Cortex Function and Structure in Depression. *Annals of the New York Academy of Sciences*.

[38] Wang T., Li S., Jiang G. (2016). Regional Homogeneity Changes in Patients With Primary Insomnia. *European Radiology*.

[39] Leerssen J., Wassing R., Ramautar J. R. (2019). Increased Hippocampal-Prefrontal Functional Connectivity in Insomnia. *Neurobiology of Learning and Memory*.

[40] Lee Y. G., Kim S., Kim N. (2018). Changes in Subcortical Resting-State Functional Connectivity in Patients With Psychophysiological Insomnia After Cognitive-Behavioral Therapy: Changes in Resting-State FC After CBT for Insomnia Patients. *Neuroimage: Clinical*.

[41] Zhang X., Yao S., Zhu X., Wang X., Zhu X., Zhong M. (2012). Gray Matter Volume Abnormalities in Individuals With Cognitive Vulnerability to Depression: A Voxel-Based Morphometry Study. *Journal of Affective Disorders*.

[42] Tubbs J. D., Ding J., Baum L., Sham P. C. (2020). Immune Dysregulation in Depression: Evidence From Genome-Wide Association. *Brain, Behavior, & Immunity - Health*.

[43] Aho V., Ollila H. M., Rantanen V. (2013). Partial Sleep Restriction Activates Immune Response-Related Gene Expression Pathways: Experimental and Epidemiological Studies in Humans. *PLoS One*.

[44] Patas K., Willing A., Demiralay C. (2018). T Cell Phenotype and T Cell Receptor Repertoire in Patients With Major Depressive Disorder. *Frontiers in Immunology*.

[45] Grosse L., Carvalho L. A., Birkenhager T. K. (2016). Circulating Cytotoxic T Cells and Natural Killer Cells as Potential Predictors for Antidepressant Response in Melancholic Depression. Restoration of T Regulatory Cell Populations After Antidepressant Therapy. *Psychopharmacology*.

[46] Mellor A. L., Munn D., Chandler P. (2003). Tryptophan Catabolism and T Cell Responses. *Advances in Experimental Medicine and Biology*.

[47] Li Y., Xiao B., Qiu W. (2010). Altered Expression of CD4(+)CD25(+) Regulatory T Cells and Its 5-HT(1a) Receptor in Patients With Major Depression Disorder. *Journal of Affective Disorders*.

[48] de Heredia F. P., Garaulet M., Gómez-Martínez S. (2014). Self-Reported Sleep Duration, White Blood Cell Counts and Cytokine Profiles in European Adolescents: The HELENA Study. *Sleep Medicine*.

[49] Schlatter J., Ortuño F., Cervera-Enguix S. (2004). Lymphocyte Subsets and Lymphokine Production in Patients With Melancholic Versus Nonmelancholic Depression. *Psychiatry Research*.

[50] Simon M. S., Arteaga-Henríquez G., Algendy A. F., Siepmann T., Illigens B. M. W. (2023). Anti-Inflammatory Treatment Efficacy in Major Depressive Disorder: A Systematic Review of Meta-Analyses. *Neuropsychiatric Disease and Treatment*.

[51] Christoffersson G., Vågesjö E., Pettersson U. S. (2014). Acute Sleep Deprivation in Healthy Young Men: Impact on Population Diversity and Function of Circulating Neutrophils. *Brain Behavior and Immunity*.

[52] Farnam A., Majidi J., Nourazar S. G., Ghojazadeh M., Movassaghpour A., Zolbanin S. M. (2016). Effect of Anger Patterns and Depression on Serum IgA and NK Cell Frequency. *Iranian Journal of Immunology*.

[53] Frank M. G., Hendricks S. E., Burke W. J., Johnson D. R. (2004). Clinical Response Augments NK Cell Activity Independent of Treatment Modality: A Randomized Double-Blind Placebo Controlled Antidepressant Trial. *Psychological Medicine*.

[54] Haack M., Serrador J., Cohen D., Simpson N., Meier-Ewert H., Mullington J. M. (2013). Increasing Sleep Duration to Lower Beat-to-Beat Blood Pressure: A Pilot Study. *Journal of Sleep Research*.

[55] Tang N., Zeng Y., He G., Chen S. (2025). Interference Between Immune Cells and Insomnia: A Bibliometric Analysis From 2000 to 2023. *Frontiers in Neurology*.

[56] Suzuki H., Savitz J., Teague T. K. (2017). Altered Populations of Natural Killer Cells, Cytotoxic T Lymphocytes, and Regulatory T Cells in Major Depressive Disorder: Association With Sleep Disturbance. *Brain, Behavior, and Immunity*.

[57] Branchi I., Poggini S., Capuron L. (2021). Brain-Immune Crosstalk in the Treatment of Major Depressive Disorder. *European Neuropsychopharmacology*.

[58] Critchley H. D., Harrison N. A. (2013). Visceral Influences on Brain and Behavior. *Neuron*.

[59] Gianaros P. J., Wager T. D. (2015). Brain-Body Pathways Linking Psychological Stress and Physical Health. *Current Directions in Psychological Science*.

[60] Marsland A. L., Kuan D. C., Sheu L. K. (2017). Systemic Inflammation and Resting State Connectivity of the Default Mode Network. *Brain Behavior and Immunity*.

[61] Faghih M., Shahraki H. R., Ghanizadeh A., Ayatollahi S. M. T. (2016). Evaluation of Simultaneous Effect of Lovastatin Plus Fluoxetine on Depression Using Linear Mixed Model With LASSO Penalty. *Global Journal of Health Science*.

[62] Chen Y., Fan A. W., Huang L. (2025). Editorial: Post-Translational Modifications in Human Cancer: Pharmacological Insights and Therapeutic Opportunities. *Frontiers in Pharmacology*.

[63] Murri M. B., Cattelani L., Chesani F., Palumbo P., Triolo F., Alexopoulos G. S. (2022). Risk Prediction Models for Depression in Community-Dwelling Older Adults. *The American Journal of Geriatric Psychiatry*.

[64] Woods A. G., Sokolowska I., Taurines R. (2012). Potential Biomarkers in Psychiatry: Focus on the Cholesterol System. *Journal of Cellular and Molecular Medicine*.

